# A preclinical micro-computed tomography database including 3D whole body organ segmentations

**DOI:** 10.1038/sdata.2018.294

**Published:** 2018-12-18

**Authors:** Stefanie Rosenhain, Zuzanna A. Magnuska, Grace G. Yamoah, Wa’el Al Rawashdeh, Fabian Kiessling, Felix Gremse

**Affiliations:** 1 Institute for Experimental Molecular Imaging, University Hospital Aachen, Forckenbeckstr. 55, 52074 Aachen, Germany; 2 Miltenyi Biotec GmbH, Friedrich-Ebert-Str. 68, 51429 Bergisch Gladbach, Germany; 3 Fraunhofer MEVIS, Institute for Medical Image Computing, Forckenbeckstr. 55, 52074 Aachen, Germany

**Keywords:** 3-D reconstruction, X-ray tomography, Image processing

## Abstract

The gold-standard of preclinical micro-computed tomography (μCT) data processing is still manual delineation of complete organs or regions by specialists. However, this method is time-consuming, error-prone, has limited reproducibility, and therefore is not suitable for large-scale data analysis. Unfortunately, robust and accurate automated whole body segmentation algorithms are still missing. In this publication, we introduce a database containing 225 murine 3D whole body μCT scans along with manual organ segmentation of most important organs including heart, liver, lung, trachea, spleen, kidneys, stomach, intestine, bladder, thigh muscle, bone, as well as subcutaneous tumors. The database includes native and contrast-enhanced, regarding spleen and liver, μCT data. All scans along with organ segmentation are freely accessible at the online repository Figshare. We encourage researchers to reuse the provided data to evaluate and improve methods and algorithms for accurate automated organ segmentation which may reduce manual segmentation effort, increase reproducibility, and even reduce the number of required laboratory animals by reducing a source of variability and having access to a reliable reference group.

## Background & Summary

Micro-computed tomography (μCT) is one of the most commonly used imaging technologies in preclinical research. It provides detailed information about the volume, textures, and abnormal alterations of internal structures in high-resolution^1–4^. Because of its high reliability and reproducibility, μCT is often used as a single imaging modality. It offers many advantages including homogenous resolution, fast acquisition, and well-calibrated voxel intensities^5–9^. In addition, other imaging modalities such as nuclear or optical imaging technologies are often combined with μCT due to the need of an anatomical reference^7,10–12^. Thus, μCT provides accurate anatomical information on the basis of its good contrast recognition especially of dense tissues such as bones or calcified structures^13–18^. The main drawback of μCT imaging is a low soft tissue contrast, which can be improved by the utilization of radiopaque contrast agents^19,20^. Nowadays, a wide range of clinical and preclinical CT contrast-enhancing agents are available. Preclinical contrast agents often show a longer blood half-life time or a more specific uptake than their clinical counterparts. Examples of them are contrast-producing lipids, iodine-containing aqueous colloids, or alkaline earth metal-based nanoparticulate contrast agents^21,22^.

When μCT scans are acquired at a low dose of X-ray, longitudinal measurements in the same animal can be performed^10,23,24^. Hence, more information per animal can be acquired and disease or treatment progression within the same animal can be determined. This leads to a reduction in the required animal number, which is in accordance with the 3 R aims (Refinement, Replacement, Reduction)^25^ for animal protection.

Nevertheless, most preclinical μCT imaging studies result in a huge amount of data that needs to be processed. Currently, the gold-standard of μCT image processing is still manual delineation of regions of interest or complete organs, although this method is laborious and limited in its reproducibility due to high user-dependence^9,26–28^. Especially in preclinical imaging studies^5,26,29–31^, the sophisticated analysis of the immense amount of μCT data is more time-consuming than the scanning procedure alone, because of the high manual effort to generate whole body organ segmentations^32^. Consequently, there is a significant need for automated segmentation tools for preclinical imaging studies.

Automated segmentation (AS) or machine-learning algorithms could address the aforementioned problems by introducing consistency, reliability, and reproducibility to the process^9,26,33–37^. Although the development of AS algorithms has gained much interest among researchers, no universal algorithm has been established yet. Multi-atlas segmentation (MAS) is one promising candidate for a new gold-standard in image annotation^26^. MAS has been successful used in both multi- and single-organ segmentations, despite the general shortcomings of abdominal imaging, i.e. shifting of organs inside the abdominal cavity. Wang et al. presented a MAS atlas dedicated to preclinical image analysis including multiple training subjects^29^. This atlas consists of 103 μCT whole body mouse images and reflects more realistically the deformation of internal organs following the changes of pose and weight due to interspecies variations and within one individual along longitudinal studies.

Nevertheless, to our knowledge no atlas or database of preclinical μCT data including organ segmentations exists, because, so far, most CT databases only include reconstructed scans or segmented bone structures^17,38^. Therefore, the aim of our study is to provide the first preclinical μCT database including whole body mouse images and their organ segmentations. Our open-access database includes 225 native and contrast-enhanced whole-animal μCT volumes along with manual organ segmentations acquired from mice scanned longitudinally in different positions. Organ parameters such as volume, surface, and distances in one individual remain stable over time. Furthermore, we calculated the Sørensen-DICE coefficient to compare the similarity between segmentations of two independent experts. This coefficient may help to compare the achieved accuracy of automated methods with the inter-user variability of manual segmentation. We highly encourage researchers to use these 3D datasets, e.g. for further comparative analysis of organ morphology or to determine relevant μCT features such as intensity or variations between voxels. Ideally, this introduced database will be used to validate segmentation and machine-learning approaches and thus, facilitate the development of reliable, simplified, and user-independent analysis tools for whole body organ segmentation. In addition, the anatomical 3D data of the whole mouse body including the main organs will serve as a visual and education resource to train researchers for segmentation of tumors and organs.

## Methods

### Datasets

**Figure 1:**
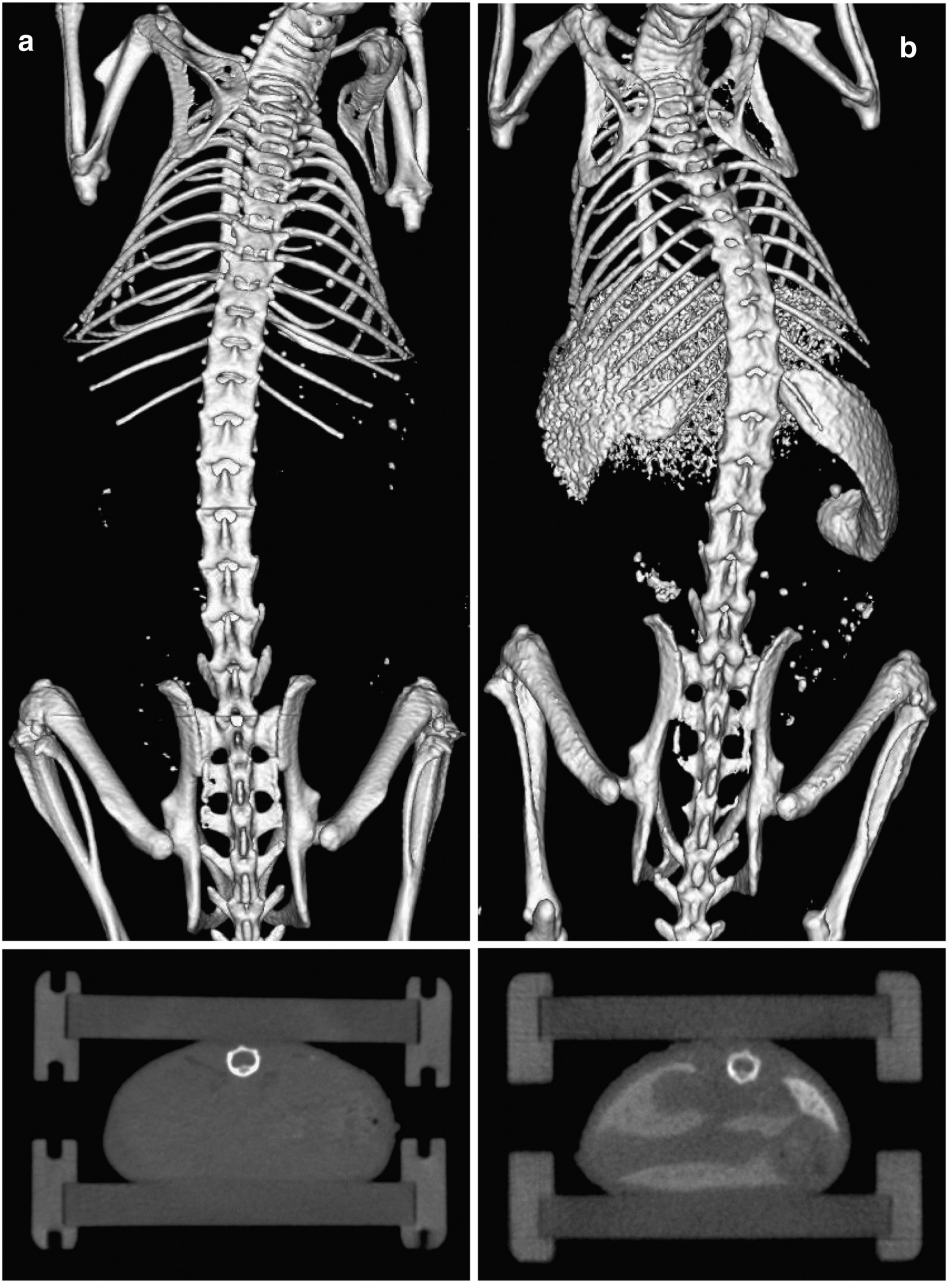
3D visualization of native and contrast-enhanced μCT data. Each μCT scan consists of a set of isotropic voxels, whereby all voxel intensities are calibrated in Hounsfield units allowing a direct comparison between native and contrast-enhanced μCT data. Using a gray scale, structures with high attenuation of X-rays appear brighter, e.g. the bones, whereas structures with low attenuation appear dark such as lung and soft tissue. (**a**) 3D rendering (upper panel) and 2D axial slice view (lower panel) for a native (#M01-0.25 h) and a contrast-enhanced scan (#M03-008h) are depicted. (**b**) Spleen and liver appear brighter after the injection of the contrast agent.

For generating this database, two μCT datasets from other studies were reused: one native dataset without using a contrast agent and one dataset with contrast-enhanced μCT scans, where the contrast agent ExiTron™ nano 6000 (Viscover, Berlin, Germany) was injected, see Fig. 1. The native μCT dataset is part of an already published study^23^. Publishing the contrast-enhanced μCT data is currently in progress. In both studies, all animal experiments were approved by the Governmental Review Committee on Animal Care. Thus, for generating this database no additional mice were required.

The native dataset includes 140 3D whole body scans acquired from 20 female BALB/c nu/nu mice (Charles River Laboratory, Sulzfeld, Germany) measured at seven time points by a preclinical μCT (Tomoscope Duo, CT Imaging GmbH, Erlangen, Germany), see Table 1. For the μCT scanning process, the mice were anesthetised using 2.5% isoflurane vaporised in 1.0 l/min of oxygen gas using a dedicated vaporiser. Afterwards, they were placed in an animal cassette as described before^10,39^. While acquiring μCT data, mice were constantly under anesthesia. For each time point; 0.25 h, 002 h, 004 h, 008 h, 024 h, 048 h, and 072 h; mice were newly anesthetised, positioned in the mouse bed, and scanned. A dual energy μCT scan (HQD-6565-360-90) was used, where tubes were operated with a voltage of 65 kV and a current of 1 mA acquiring 720 projections with 1032 × 1012 pixels during one full rotation, respectively as it was described in detail before^10,39,40^. Per scan a time of 90 s was required, whereby two scans per mouse were needed at each time point to entirely cover the mouse body. The acquired voxel sizes were 0.28 mm × 0.28 mm × 0.28 mm and the field of view was 40.32 mm × 28.84 mm × 55.44 mm. The spatial resolution of the system is in the order of 80 μm with a fixed geometry.

The contrast-enhanced dataset consists of 85 3D whole body scans from ten female A431-tumor bearing BALB/cAnNRj-Foxn1nu mice (Janvier, Le Genest-Saint-Isle, France), see Table 2. They were scanned with the InSyTe μCT scanner (BMIF TriFoil Imaging, Dijon, France). One hour before the first scan, the preclinical μCT contrast agent ExiTron™ nano 6000 (100 μl, 640 mg iodine/kg body weight) was intravenously injected. This non-toxic, commercially available, alkaline earth metal-based nanoparticulate contrast agent circulates in the blood stream and is taken up by the Kupffer cells. It significantly enhances the CT-contrast in spleen and liver^21,41^ as clearly shown in Fig. 1. A single dose of ExiTron™ nano 6000 results in longstanding enhancement of liver and spleen tissue for longer than 3 weeks peaking for the liver at approximately 4 h and for spleen contrast at 48 h post injection^41^. For scanning procedure, the mice were anaesthetised in the same way and placed in the same animal cassette as described in the case of the native dataset. A special adapter was designed and built for this μCT. Hence, the same mouse bed from the previous study was used among the different μCT systems in order to increase the consistency of μCT analysis. Similar to the protocol of the native μCT scans, the mice were repeatedly anesthetised, positioned in the mouse bed, and scanned at the different time points; pre (−001h), 0.25 h, 002 h, 004 h, 006 h, 008 h, 024 h, 048 h, 072 h, 144 h, 168 h, 192 h, and 240 h. For a full-rotation μCT scan, 207 views with a frame rate of 1 frame per view, an X-ray tube voltage of 75 kV, and an exposure time of 230 ms were acquired. The acquired voxel sizes were 0.28 mm × 0.28 mm × 0.28 mm and the field of view was 43.12 mm × 33.88 mm × 67.76 mm.

### Image reconstruction and analysis - 3D whole body organ segmentation

**Figure 2:**
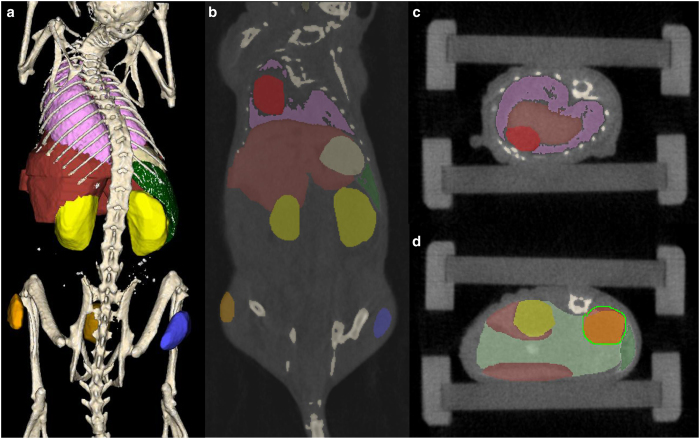
Interactive organ segmentation based on whole body μCT data. (**a**–**d**) A μCT-based 3D whole body organ segmentation of a mouse is performed semi-automatically for bones (beige), lung (pink), and spleen (dark green). Other organs are segmented by manual delineation: liver (brown), stomach (light tan), kidneys (yellow), intestine (light green, only depicted in d), tumor (orange), part of the thigh muscle (blue), and bladder (gold). For the segmentation process, organs need to be encircled in several slices such as (**b**) coronal and (**c**) axial from which a program can interpolate the remaining slices. (**d**) Example of the manual delineation procedure by drawing scribbles (green line) around the right kidney. Mouse #M03-004h of the contrast-enhanced dataset was used in this example.

All acquired 3D μCT images were reconstructed at an isotropic voxel size of 28 μm using a Feldkamp type algorithm and a smooth kernel as previously described^10,23,39^. 3D organ segmentations based on the μCT data were performed for all mice at the different time points. The standardised segmentation protocol, used for both datasets, was developed in our group and has been previously described^42^. Briefly, bone structures and lung were semi-automatically segmented using threshold functions above a certain value, for bone >1000 HU, or below a certain value, for lung <300 HU, and selecting a seed point for region growing. Organs with defined and clearly visible boundaries such as the heart, bladder, and kidneys were segmented by manual delineation. Scribbles were drawn around the organ boundaries, see Fig. 2d. Other organs such as the stomach and intestine were segmented approximated by a few convex regions and manual delineation of them. Liver segmentation was performed slab wise due to the complex shape of the lobes. As an example of muscle, a part of the thigh was segmented. Despite their polymorphic shape subcutaneous tumors displayed clearly distinguishable boundaries and were segmented by manual delineation.

### Statistics and calculation of the Sørensen–Dice coefficient

The quality of the whole body organ segmentations by manual delineation between two trained scientists was compared by calculating the Sørensen–Dice coefficient (Sørensen index, Dice’s coefficient). This similarity coefficient is widely used in image analysis, for example, to evaluate the reproducibility of manual segmentations and the overlap accuracy of automated probabilistic fractional segmentation of MR images^28,43^. Here in particular, it is used to investigate the similarity between the same organ analysed independently by two experts. The Sørensen-Dice similarity coefficient for image segmentation is calculated using this formula:

$$sv=\frac{2|X\cap^{\;}Y|}{\left|X|+|Y\right|}$$

For each particular organ, X and Y represent the set of segmented voxels of user 1 and 2, respectively. The Sørensen–Dice coefficient computes the ratio of segmentation overlap to the segmentation size. A higher Sørensen–Dice coefficient represents a higher degree of similarity. A score of 1.0 denotes a perfect overlap and a score of 0.0 represents no overlap. Thus, the Sørensen–Dice coefficient can be used to determine the accuracy of automated segmentation methods by comparison with manual segmentations.

**Figure 3:**
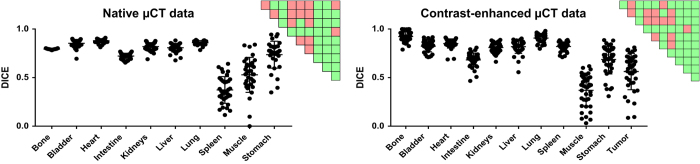
Analysis of the Sørensen–Dice coefficient including pair-wise significance matrices. The highest variability occurs for the spleen in the native dataset due to the low soft tissue contrast of μCT images (DICE of 0.373) as well as for muscle (native: 0.528 and contrast-enhanced: 0.369), intestine (0.722 and 0.682), and stomach (0.736 and 0.686) for both datasets. All other organs, especially those with clear organ boundaries such as bladder and heart, depict a good Sørensen-Dice coefficient, nearly reaching the optimum 1.0 (=perfect overlap). Statistical significances are shown as pair-wise significance matrices (p &lt; 0.05 in green). The matrices demonstrate that analysing the native μCT data, the highest user-dependent errors occur for spleen, muscle, and stomach. For the contrast-enhanced μCT data, the highest user-dependent errors occur in segmenting muscle, stomach, and tumor.

The Sørensen–Dice coefficient was computed for both datasets and all segmented organs to assess inter-user segmentation variability, see Table 3. For the native dataset, 35 whole body μCT-based organ segmentations were performed by a second evaluator. All mice that received the fluorescent probe OsteoSense 750 EX (PerkinElmer, USA) at all seven time points were chosen for this analysis^23^, see Table 1. This probe has no decreasing or enhancing effect on CT-contrast. For the contrast-enhanced μCT dataset, 39 organ segmentations were used for calculating the Sørensen-Dice coefficient. All eight mice, but only the time points 0.25 h, 002 h, 004 h, 006 h, and 008 h were chosen for this analysis, see Table 2. Time point 008 h of #M01 is missing due to some technical problems during the scanning process. Statistical analysis was performed using GraphPad Prism version 7.0. For the comparison between organs, a multi-comparison one-way ANOVA was performed in combination with a Tukey posttest. A p-value below 0.05 was considered to represent statistical significance. Statistical significances are shown as pair-wise significance matrices (P &lt; 0.05 in green) in Fig. 3, detailed explanation has been previously described^23^.

## Data Records

The μCT database published in this article consists of native and contrast-enhanced μCT scans. The native dataset comprises 140 murine 3D whole body scans and organ segmentations, where 35 scans include organ segmentations from two different evaluators. The contrast-enhanced dataset includes 85 murine 3D whole body scans with enhanced contrast in spleen, liver, and other organs, where 39 scans include two organ segmentations. Both datasets have been deposited in an online Figshare repository (Data Citation 1). For each scan, there is a subfolder labeled with mouse ID (M01, M02, etc.) and time point of measurement (0.25 h, 002 h, etc.) which contains a pyramid of μCT data with different resolutions (CT140, CT280) in the Analyze file format (consisting of pairs of .HDR and .IMG files). CT280 is generated by averaging eight neighboring voxels of CT140 to one average voxel, which results in a lower resolution. For the organ segmentations of the native data, the CT280 scan was used. The CT140 scans were initially used for the segmentations of the contrast-enhanced data, but, additionally, the organ segmentations were saved using CT280, clearly marked in the file names (Organ_140 or Organ_280). All 3D organ segmentations are saved as Analyze files with 8-bit voxels containing different indices for each segmented organ. Every voxel belongs exactly to one class index, either to an organ class or to class 0 (unclassified). The folder also includes a text file ending with .CLS, describing the assignment of the class indices to the respective organ and class color, for example: ClassColors = 0 0 0 255|201 238 255 255|255 170 255 255, ClassIndices = 0|1|2, ClassNames = unclassified|Bone|Lung. Additionally, a segmentation file named *Bed including the mouse bed, the whole body of the mouse, and fiducial markers, is included in every folder.

## Technical Validation

The intensity values of μCT images are usually provided in Hounsfield units, which are calibrated in such a way that air generates intensities of −1000 and water 0. Therefore all CT images acquired by different scanners can be compared with each other due to the general calibration. Both preclinical μCT scanners were regularly maintained including calibration and quality control under the responsibility of qualified service personnel from the respective companies. However, occurring image artifacts, ring or beam-hardening artifacts, or motion artifacts due to breathing or cardiac movements can result in discrepancies between reconstructed values and true attenuation coefficients. In our study, these artifacts are negligible, because the manual segmentation is not influenced by any kind of artifacts, because when organ segmentation by manual delineation is performed, most organ boundaries can be seen by eye even if they are blurred. Nevertheless, the artifacts might interfere with some automated organ segmentation algorithms under certain conditions and should be considered in detail. Furthermore, the used multimodal mouse bed places the animal in a fixed position which leads to a reduction of breathing and motion artefacts. This mouse cassette is routinely used in many research institutes and companies, for several applications such as FMT-CT, PET-CT.

## Usage Notes

Researchers are highly encouraged to download the 3D μCT scans of the native and/or contrast-enhanced μCT datasets from Figshare (Data Citation 1). The μCT data including organ segmentations could be used for the development of automated organ segmentation algorithms. By computing the Sørensen-DICE coefficient, the accuracy of existing or newly developed approaches can be compared. Usage of the well-known Analyze file format ensures that the μCT data can be loaded by many 3D analysis software packages. For all analysis, we used the software “Imalytics Preclinical”^42^, which was developed in our group.

## Additional information

**How to cite this article**: Rosenhain, S. *et al*. A preclinical micro-computed tomography database including 3D whole body organ segmentations. *Sci. Data*. 5:180294 doi: 10.1038/sdata.2018.294 (2018).

**Publisher’s note**: Springer Nature remains neutral with regard to jurisdictional claims in published maps and institutional affiliations.

## Supplementary Material



## Figures and Tables

**Table 1:** Characterization of the native dataset.

Source	Mouse ID	Temporal range (bold: 2 organ segmentations are available)
native	M01	**0.25 h; 002 h; 004 h; 008 h; 024 h; 048 h; 072 h**
native	M02	**0.25 h; 002 h; 004 h; 008 h; 024 h; 048 h; 072 h**
native	M03	**0.25 h; 002 h; 004 h; 008 h; 024 h; 048 h; 072 h**
native	M04	**0.25 h; 002 h; 004 h; 008 h; 024 h; 048 h; 072 h**
native	M05	**0.25 h; 002 h; 004 h; 008 h; 024 h; 048 h; 072 h**
native	M06	0.25 h; 002 h; 004 h; 008 h; 024 h; 048 h; 072 h
native	M07	0.25 h; 002 h; 004 h; 008 h; 024 h; 048 h; 072 h
native	M08	0.25 h; 002 h; 004 h; 008 h; 024 h; 048 h; 072 h
native	M09	0.25 h; 002 h; 004 h; 008 h; 024 h; 048 h; 072 h
native	M10	0.25 h; 002 h; 004 h; 008 h; 024 h; 048 h; 072 h
native	M11	0.25 h; 002 h; 004 h; 008 h; 024 h; 048 h; 072 h
native	M12	0.25 h; 002 h; 004 h; 008 h; 024 h; 048 h; 072 h
native	M13	0.25 h; 002 h; 004 h; 008 h; 024 h; 048 h; 072 h
native	M14	0.25 h; 002 h; 004 h; 008 h; 024 h; 048 h; 072 h
native	M15	0.25 h; 002 h; 004 h; 008 h; 024 h; 048 h; 072 h
native	M16	0.25 h; 002 h; 004 h; 008 h; 024 h; 048 h; 072 h
native	M17	0.25 h; 002 h; 004 h; 008 h; 024 h; 048 h; 072 h
native	M18	0.25 h; 002 h; 004 h; 008 h; 024 h; 048 h; 072 h
native	M19	0.25 h; 002 h; 004 h; 008 h; 024 h; 048 h; 072 h
native	M20	0.25 h; 002 h; 004 h; 008 h; 024 h; 048 h; 072 h
The table shows the details of the native dataset, mouse IDs, measured time points, and the time points where 2 organ segmentations are available.		

**Table 2:** Characterization of the contrast-enhanced dataset.

Source	Mouse ID	Temporal range (bold: 2 organ segmentations are available)
Contrast-enhanced	M01	−001h**; 002 h; 004 h; 006 h;** 024 h; 048 h; 072 h; 120 h; 168 h; 240 h
Contrast-enhanced	M02	**0.25 h; 002 h; 004 h; 006 h; 008 h;** 024 h
Contrast-enhanced	M03	−001h; **0.25 h; 002 h; 004 h; 006 h; 008 h**; 024 h; 048 h; 072 h
Contrast-enhanced	M04	−001h; **0.25 h; 002 h; 004 h; 006 h; 008 h**; 024 h; 048 h; 072 h; 120 h; 144 h; 192 h; 240 h
Contrast-enhanced	M05	−001h; **0.25 h; 002 h; 004 h; 006 h; 008 h**; 024 h; 048 h; 072 h
Contrast-enhanced	M06	−001h; **0.25 h; 002 h; 004 h; 006 h; 008 h**; 024 h; 048 h; 072 h; 120 h; 144 h; 192 h; 240 h
Contrast-enhanced	M07	−001h; **0.25 h; 002 h; 004 h; 006 h; 008 h**; 024 h; 048 h; 072 h; 120 h
Contrast-enhanced	M08	−001h; **0.25 h; 002 h; 004 h; 006 h; 008 h**; 024 h; 048 h; 072 h; 120 h; 144 h; 168 h; 192 h
Contrast-enhanced	M09	024 h
Contrast-enhanced	M10	024 h
The table shows the details of the contrast-enhanced dataset, mouse IDs, measured time points, and the time points where 2 organ segmentations are available.		

**Table 3:** Comparison of the Sørensen–Dice coefficient.

Native μCT data					Contrast-enhanced μCT data			
Organ	Dice	Std dev	Minimum	Maximum	Dice	Std dev	Minimum	Maximum
Bone	**0.793**	0.005	0.782	0.803	**0.923**	0.050	0.789	0.999
Bladder	**0.854**	0.039	0.694	0.900	**0.822**	0.057	0.710	0.915
Heart	**0.879**	0.021	0.812	0.910	**0.851**	0.047	0.687	0.913
Intestine	**0.722**	0.029	0.654	0.768	**0.686**	0.149	0.308	0.886
Kidneys	**0.819**	0.040	0.689	0.878	**0.809**	0.051	0.662	0.888
Liver	**0.808**	0.044	0.677	0.883	**0.818**	0.068	0.555	0.903
Lung	**0.859**	0.021	0.784	0.888	**0.907**	0.048	0.797	0.980
Spleen	*0.373	0.137	0.115	0.642	*0.820	0.046	0.710	0.888
Muscle	**0.528**	0.179	0	0.839	**0.369**	0.162	0.031	0.618
Stomach	**0.736**	0.138	0.348	0.947	**0.682**	0.070	0.466	0.809
Tumor	**-**	**0.562**	0.187	0.087	0.810			
The coefficients for all organs of the native and the contrast-enhanced datasets are depicted to assess the quality of two organ segmentations by manual delineation. Furthermore, the calculated standard deviation (Std dev) and the minimum and maximum values of the Sørensen–Dice coefficient are shown. The main difference between the native and the contrast-enhanced μCT data is the increase in Sørensen–Dice coefficient showing the higher similarity in segmentation of the spleen (increase from 0.373 to 0.820, as highlighted with *). The data are also graphically depicted in Fig. 3.								
